# Generational Shifts in Adolescent Mental Health: A Longitudinal Time-Lag Study

**DOI:** 10.1007/s10964-024-02095-3

**Published:** 2024-10-12

**Authors:** Meghan E. Borg, Taylor Heffer, Teena Willoughby

**Affiliations:** 1 Brock University, St. Catharines, ON Canada; 2 Ontario Tech University, Oshawa, ON Canada

**Keywords:** adolescence, mental health, longitudinal, generational

## Abstract

There is concern that adolescents today are experiencing a “mental health crisis” compared to previous generations. Research has lacked a longitudinal time-lag design to directly compare depressive symptoms and social anxiety of adolescents in two generations. The current study surveyed 1081 adolescents in the current generation (*M*age = 14.60, *SD* = 0.31, 49% female) and 1211 adolescents in a previous generation (*M*age = 14.40, *SD* = 0.51, 51% female) across the high school years (grades 9–12), 20 years apart. Mixed-effects analysis revealed that the Current-Sample reported higher and increasing mental health problems over time compared to the Past-Sample. Although most adolescents reported consistently low mental health problems, the Current-Sample had a higher proportion of adolescents who were consistently at risk across the high school years compared to the Past-Sample. These findings highlight while most adolescents in both generations do not report elevated mental health problems, there may be a small, yet growing, group of adolescents today at risk for experiencing a “mental health crisis”.

## Introduction

There has been tremendous concern from researchers, parents, practitioners, and media about the mental health of adolescents today, with some suggesting that adolescents today are uniquely facing a “mental health crisis” (Abbasi, 2023; Aftab & Druss, 2023; American Academy of Pediatrics, 2021; Benton et al., 2021; Duffy et al., 2019; Madigan et al., 2023; Panchal et al., 2023; Racine, Hetherington, et al., 2021; Racine, McArthur, et al., 2021; Shoshani & Kor, 2022). To test this suggestion, several researchers have assessed generational mental health differences cross-sectionally (Collishaw et al., 2010; Patalay & Gage, 2019; Twenge, 2011) and found, on average, that adolescents today are reporting higher mental health problems than adolescents from earlier cohorts. It is important, however, also to longitudinally investigate mental health over time *within* each generational cohort to identify if mental health trajectories during adolescence are consistent between generations. Further, past studies have not investigated whether there are generational differences in the *consistency* of adolescent mental health problems over time. For example, some adolescents may have persistent difficulties with mental health over time whereas others may face more situational mental health problems that occur only at one time point. The goal of the current study is to address this gap in the literature by using a longitudinal time-lag design to examine trajectories and consistency of depressive symptoms and social anxiety over time across two generations of adolescents from the same high schools, 20 years apart.

### Evidence of a “Mental Health Crisis” During Adolescence?

Adolescent mental health is an important global public health issue. Theories of adolescent development propose that adolescents may have unique vulnerability to mental health problems (Kim, 2020). Specifically, adolescence is thought to be a period of enhanced reactivity to stressful and emotional situations (likely related to puberty) during a time when their capacity for cognitive control is still developing (Blakemore & Mills, 2014; Casey, 2015; Foulkes & Blakemore, 2016; Willoughby et al., 2021). Thus, it is critical to investigate whether adolescents today may be even more at risk for mental health problems compared to earlier generations (Blakemore, 2019; Towner et al., 2023). Without a doubt, the COVID-19 pandemic has exacerbated the concern of a youth mental health crisis, resulting in many recent studies focusing on the impact of the pandemic on adolescent mental health (Afriat et al., 2023; De France et al., 2021; Garagiola et al., 2022; Houghton et al., 2022; Loades et al., 2020; Wolf & Schmitz, 2023). Research findings indicate, however, that the presence of elevated mental health problems started prior to the beginning of the pandemic (Benton et al., 2021; Lebrun-Harris et al., 2022; Schweizer et al., 2023; Slobodskaya et al., 2022), suggesting that there may be other, potentially generational, factors at play.

To effectively understand whether the current generation of adolescents (often referred to as “Generation Z” or “iGen”, born between 1997 and 2010) (Dimock, 2019) indeed are experiencing a unique mental health crisis, it is necessary to directly compare levels of mental health problems to a previous generation. Several studies have used a time-lag cross-sectional design, which is the ideal design for examining time period and birth cohort differences (Schaie, 1965). These studies found support for higher depressive symptoms, suicide ideation, anxiety, and other internalizing problems among more recent adolescent cohorts compared to past adolescent cohorts (Collishaw et al., 2010; Fleming et al., 2022; Hagquist, 2009; Parodi et al., 2021; Twenge, 2011, 2015; Weinberger et al., 2018; Xin et al., 2010), whereas other studies have found no evidence of differences in internalizing mental health problems between previous and current cohorts (Cosma et al., 2020) (see Bor et al., 2014 for a systematic review). Importantly, studies assessing sex differences found on average, higher internalizing problems among adolescent girls in recent cohorts compared to previous cohorts (Daly, 2022; Morken et al., 2023), whereas findings among adolescent boys were mixed. Of note, many of these studies focused on comparisons between adolescents from the 1990s compared to adolescents in 2005 to 2012. More contemporary evidence is needed to develop a comprehensive body of evidence regarding these generational effects. The current study addresses this gap in the literature.

### The Value of Using Longitudinal Data to Address Concerns of a “Mental Health Crisis”

The majority of research assessing generational differences in mental health problems typically used concurrent cross-sectional data. As such, these studies do not allow for an investigation of change (or stability) in mental health problems *within* each generational cohort, in addition to differences in mental health problems *between* each generational cohort. A longitudinal design allows for the consideration of both change within an individual as well as differences between generations.

One way to assess change (or stability) of mental health problems within adolescents is to look at consistency of mental health problems across time points. For example, an adolescent may report elevated mental health problems at one time point, but these problems may not persist over time; that is, these mental health problems may be situational and not indicative of a chronic problem (Supke et al., 2021). Other adolescents, however, may report elevated mental health problems at multiple time points, which may indicate that they are at risk for ongoing mental health problems that would require access to different resources and interventions than adolescents experiencing situational mental health problems. If mental health problems are assessed only at one time point, adolescents who experience situational and chronic mental health problems are conflated into one “at-risk” group, which may not paint an accurate picture of the ways in which adolescents experience mental health problems.

The suggestion of an “adolescent mental health crisis” implies that most adolescents today, *in general*, are experiencing elevated mental health problems compared to a previous generation. There may be, however, multiple reasons for generational differences in average mental health problems. It may be that indeed many adolescents today are experiencing higher levels of mental health problems compared to a previous generation. Or it may be that there is a smaller, yet growing, proportion of adolescents today who experience substantially higher mental health problems (situational and/or chronic), which inflates the overall average. Testing these potential explanations is a critical contribution to the literature on the adolescent mental health crisis, as it may provide insights for how to identify and target adolescents who are most at risk for mental health problems over time.

## Current Study

Previous research has lacked a longitudinal time-lag comparison of adolescents from two generations to assess whether adolescents today report worse mental health over time and are more consistently at risk for elevated mental health problems compared to adolescents from a previous generation. The purpose of the current study is to examine trajectories and consistency of mental health problems over time across two generations of adolescents from the same high schools. The following research questions are addressed: Are there generational differences in mental health trajectories across the high school years (Research Question 1)? Are there generational differences in the proportion of adolescents at risk for consistently elevated mental health problems (Research Question 2)? Consistent with previous research, it was hypothesized that on average, the current generation would report higher mental health problems than the previous generation. Given the lack of research assessing consistency in mental health problems within generations, it is unclear whether the generations will differ in their consistency of elevated mental health problems over time; thus, these analyses were exploratory.

## Method

### Participants

Participants were drawn from the same two high schools in Ontario, Canada, as part of two separate larger longitudinal-sequential studies on health-risk behaviors and youth lifestyle choices, 20 years apart. The average level of parental education (used as a proxy for socioeconomic status) in both samples was between some university/college and an associate degree/diploma, suggesting that both samples represent a middle-class population (Census in Brief: Does education pay? A comparison of earnings by level of education in Canada and its provinces and territories, 2017).

The Past-Sample involved 1211 participants (*M*age = 14.40, *SD* = 0.51, 51% female) who entered the study either in 2003 (24%; all were in grade 9), 2004 (41% were in grade 9 and 27% were in grade 10), or 2006 (8%; all were in grade 10). Participants completed the survey annually until they were in grade 12.

The Current-Sample consisted of 1081 students (*M*age = 14.60, *SD* = 0.31, 49% female) who were surveyed annually beginning in 2017. Participants were in grades 3 to 8 at the beginning of the study in 2017; thus, participants started high school in different years of the study. To be consistent with the Past-Sample, the Current-Sample was restricted to only include data when participants were in high school. Participants entered grade 9 in 2018 (7.21%), 2019 (22.27%), 2020 (22.69%), 2021 (24.18%), or 2022 (23.65%). As the last year of data collection for the Current-Sample was in 2022, participants who entered grade 9 in 2020, 2021, or 2022 were not able to complete the survey in grade 12, grades 11–12, or grades 10–12 respectively (see missing data section for more information).^1^

### Procedure

In the Past-Sample, active informed assent was obtained from participants. A letter outlining the study was mailed to the parents at each student’s home prior to the survey administration. The letter indicated that parents could request that their adolescent not participate in the study. An automated phone message about the study also was left at each student’s home phone number. This procedure was approved by the participating school board and the university Research Ethics Board. The survey was administered each year to students in classrooms by trained research staff. Participants completed the survey by hand in their classrooms and received small gifts as compensation. Students were informed that their responses were completely confidential. The survey took approximately one hour to complete.

In the Current-Sample, parents provided informed consent and participants provided informed assent before completing the survey. This study was approved by the University Research Ethics Board. The survey was administered in Years 1 to 3 (2017–2019) to students in classrooms by trained research staff. In Years 1 and 2, participants completed the survey by hand in their classrooms. In Year 3, participants completed the survey online through Qualtrics on tablets in their classrooms. In Years 4 to 6 (2020–2022), students completed the survey online through Qualtrics at home due to school restrictions given the COVID-19 pandemic. Participants received small gifts as compensation. Students were informed that their responses were completely confidential. The survey took approximately one hour to complete.

### Missing Data

Missing data occurred each year because some students who were present for the survey did not answer all survey questions (average missing data ranged from 5% to 12% across the high school years in the Past-Sample, and from 1% to 3% across the high school years in the Current-Sample) and because some students did not complete the survey, either because they were not invited to complete the survey (35% of the Past-Sample were not invited to complete the survey in grade 9 due to school restrictions) or because of absenteeism (ranging from 15% to 34% across the high school years in the Past-Sample and 19% to 27% in the Current-Sample for Years 1 to 3 of the study). Absenteeism from class was due to illness, a co-op placement, a free period, or involvement in another school activity. In Years 4 to 6 for the Current-Sample, surveys were conducted online at home because of pandemic restrictions; missingness in this case was due to students not completing the survey when invited to do so or due to having no contact information (average missing data ranged from 38% to 49% in these years of the study). Missing data were imputed separately for each sample using the expectation-maximization (EM) algorithm with all study variables and demographics included in the analyses. EM retains cases that have missing data, thus avoiding the biased parameter estimates that can occur with pairwise or listwise deletion (Schafer & Graham, 2002). Past research has demonstrated that methods of multiple imputation can produce unbiased results even with large proportions of missing data (Madley-Dowd et al., 2019). Note, participants who entered grade 9 later in the study (i.e., in 2020, 2021, or 2022) did not have the opportunity to complete the survey in the later years of high school as they were too young. In that case, values were not imputed for the missing grades as these data are incomplete rather than missing. Contemporary analysis techniques can handle incomplete data, so imputation is not necessary.

### Measures

All measures were consistent across the samples.^2^

#### Demographics

Grade, sex (male, female), and parental education (one item per parent/caregiver using a scale from 1 = *did not finish high school* to 6 = *completed a professional or graduate degree*, which was then averaged across parents/caregivers; used as a proxy for SES) were assessed.

#### Depressive symptoms

Depressive symptoms were measured using 13 items from the Center for Epidemiologic Studies Depression Scale (Weissman et al., 1980). Participants indicated how often they experienced symptoms of depression over the past two weeks. Response options for these items (e.g., “I felt sad”, “I was bothered by things that usually don’t bother me”) ranged from 1 = *not at all* to 4 = *a lot of the time*. Greater scores were indicative of higher levels of depressive symptoms. Cronbach’s alphas for this scale at each year ranged from 0.85 to 0.88 in the Past-Sample and from 0.87 to 0.90 in the Current-Sample.

#### Social anxiety

Social anxiety was measured using four items from the Social Anxiety Scale for Children-Revised (La Greca & Stone, 1993). These items (e.g., “I am quiet when I am with a group of other students my age”, “I am afraid that other people my age will not like me”) were measured on a 4-point Likert scale ranging from 1 = *almost never* to 4 = *almost always*. Higher scores indicated higher levels of social anxiety. Cronbach’s alphas for this scale at each year ranged from 0.70 to 0.74 in the Past-Sample and from 0.70 to 0.79 in the Current-Sample.

### Plan of Analysis

First, preliminary analyses were conducted to assess whether there were any differences in depressive symptoms and social anxiety in years during compared to outside of the pandemic in the Current-Sample. Specifically, these analyses were conducted to ensure that any differences detected between the Past-Sample and Current-Sample were not driven by changes related to the pandemic. To address this question, two mixed-effects models were run with depressive symptoms and social anxiety as the dependent variables. For each model, the main effects of grade (recoded as 0 to 3) and pandemic year (a factor that was dummy coded as 0 and 1, indicating whether each year of the study was a pandemic year [2020 or 2021] or not a pandemic year) were included, as well as an interaction between these two variables. Sex and SES were controlled for, and each model included a random intercept for subject nested within cohort to account for the longitudinal and nested nature of the data. A Bonferroni correction was applied to account for two models (*p* < 0.025).

Next, to examine whether the Past-Sample differed from the Current-Sample on depressive symptoms and social anxiety across the high school grades (Research Question 1), two mixed-effects models were run. The indicators of mental health were included as the dependent variables in these models. For each model, the main effects of grade and sample (a factor that was dummy coded as 0 and 1, indicating whether adolescents were in the Past-Sample or the Current-Sample), were included in the models, as well as an interaction between these two variables. Sex and SES were controlled for, and each model included a random intercept for subject nested within sample cohorts to account for the longitudinal and nested nature of the data. These models were implemented using the *lmerTest* package (v 1.8.42) (Kuznetsova et al., 2017) in R (v 4.3.1) (R Core Team, 2023). The “plot_model” function from the *sjPlot* package (v 2.8.15) (Lüdecke et al., 2023) was used to visualize effects from the model. A Bonferroni correction was applied to account for two models (*p* < 0.025).

In a follow-up analysis, sex differences in mental health trends across high school between the two generations of adolescents were examined. Two mixed-effects regressions were conducted. The indicators of mental health were included as the dependent variables in these models. For each model, the main effects of grade, sex (a factor that was dummy coded as 0 and 1, indicating whether adolescents were male or female), and sample (a factor that was dummy coded as 0 and 1, indicating whether adolescents were in the Past-Sample or the Current-Sample), were included in the models, as well as each 2-way interaction and the 3-way interaction between these variables. SES was controlled for, and each model included a random intercept for subject nested within sample cohorts to account for the longitudinal and nested nature of the data. These models were implemented using the *lmerTest* package (v 1.8.42) (Kuznetsova et al., 2017) in R (v 4.3.1) (R Core Team, 2023). A Bonferroni correction was applied to account for two models (*p* < 0.025).

Finally, to examine whether the Past-Sample differed from the Current-Sample on the proportion of adolescents who were consistently at-risk for elevated mental health problems (Research Question 2), a binomial regression was conducted for each mental health indicator. This analysis models differences between the two samples in the number of time points that an adolescent is considered at risk (i.e., a score >= 2.5, equivalent to experiencing symptoms of each mental health indicator “often or more”) across the number of total time points that they participated in the study. Adolescents who entered grade 9 later in the study (i.e., in 2020, 2021, or 2022) did not have the opportunity to complete the survey in the later years of high school as they were too young; thus, simply counting the number of times these participants experienced elevated mental health problems would not adequately capture how consistently they were at risk. Given that this research question addressed consistency over time, only adolescents who had the opportunity to complete the survey more than once were included in this analysis; that is, 224 participants were removed from the Current-Sample for this analysis because they did not have the opportunity to complete the survey more than once. No participants were removed from the Past-Sample for this analysis. Sex and SES were entered as control variables. This model was implemented using the “glm” function from the *stats* package (v.4.3.1) in R (v 4.3.1) (R Core Team, 2023). A Bonferroni correction was applied to account for two models (*p* < 0.025).

## Results

**Fig. 1:**
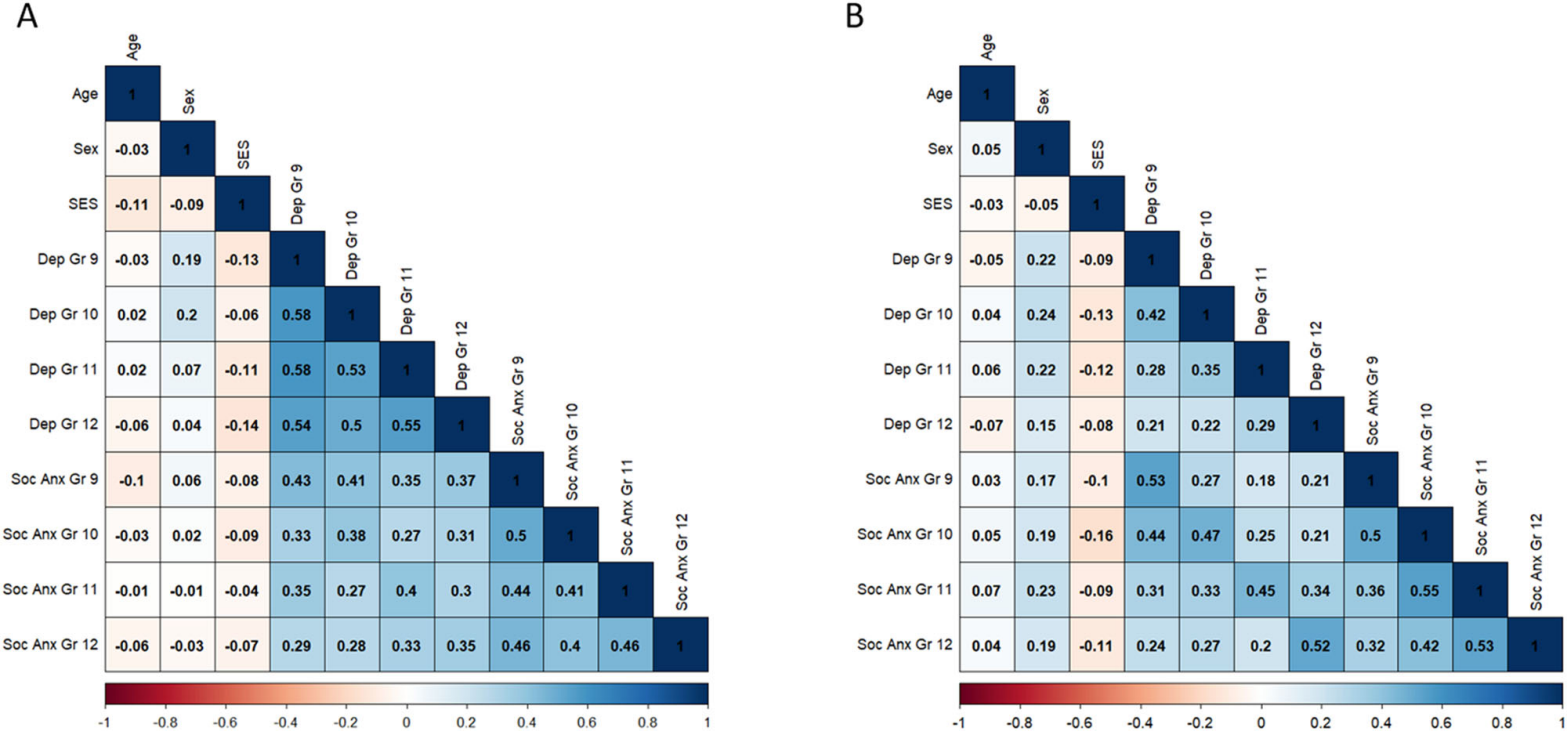
Correlations among study variables. Note. Correlations among study variables are shown in (**A**) the Past-Sample and (**B**) the Current-Sample. Age, age at year 1; Dep, Depressive symptoms; Soc Anx, Social anxiety

Descriptive statistics (means and standard deviations) are shown in Table 1. Of note, the average levels of depressive symptoms and social anxiety across all high school years in both samples were around a two on each respective scale, indicating that adolescents experienced symptoms of mental health problems “sometimes”. Correlations among study variables are shown in Fig. 1.

**Table 1:** Descriptive statistics for study variables

	Past-Sample (*n* = 1211)		Current-Sample (*n* = 1081)	
	Mean	SD	Mean	SD
Demographics				
Age (in grade 9, year 1)	14.41	0.51	14.63	0.31
Sex (% female)	48.6%	-	51%	-
Parental education	3.23	0.98	4.19	0.75
Depressive Symptoms				
Grade 9	1.50	0.32	1.88	0.47
Grade 10	1.52	0.40	1.93	0.42
Grade 11	1.55	0.44	1.99	0.45
Grade 12	1.58	0.41	2.04	0.45
Social Anxiety				
Grade 9	1.71	0.41	1.92	0.55
Grade 10	1.73	0.49	1.96	0.51
Grade 11	1.72	0.51	2.02	0.55
Grade 12	1.66	0.42	2.02	0.51

Adolescents in the past-sample and the current-sample were from the same high schools, 20 years apart

### Preliminary Analyses

**Fig. 2:**
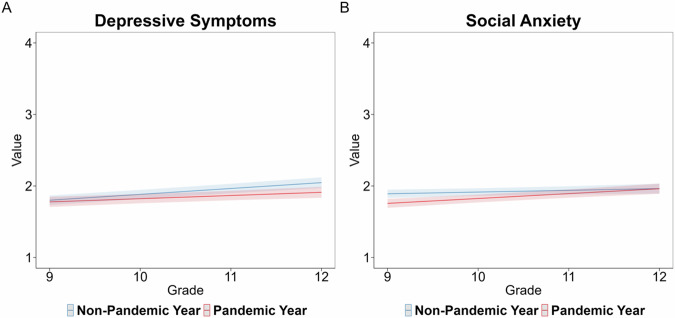
Effects plots for linear mixed-effects models for pandemic-related differences. Note. Effects plots for pandemic-related differences in depressive symptoms (**A**) and social anxiety (**B**) in the Current-Sample. The solid-colored lines represent the model fit. The shaded color regions depict the 95% confidence interval (CI)

Two mixed effects regression analyses to test for pandemic effects were conducted for depressive symptoms and social anxiety. For depressive symptoms, there was no significant main effect of the pandemic (*B* = −0.024, *SE* = 0.027, *p* = 0.380) or significant interaction between the pandemic and grade (*B* = −0.037, *SE* = 0.018, *p* = 0.036; note that this effect is trending after correcting for multiple comparisons). For social anxiety, there was a significant main effect of the pandemic (*B* = −0.134, *SE* = 0.028, *p* < 0.001) and interaction between the pandemic and grade (*B* = 0.045, *SE* = 0.019, *p* = 0.019). The interaction indicated that social anxiety was higher in grade 9 during non-pandemic years compared to pandemic years, but this difference became smaller over the high school years. See Fig. 2 for the effects plots depicting the interactions. Given that these pandemic-related differences were minimal and indicated that mental health was not worse during the pandemic, all years of the study were included to assess differences in the mental health indicators from grade 9 to grade 12 (to be consistent with the Past-Sample).

**Research question 1: Are there generational differences in mental health trajectories across the high school years?**

Full parameter estimates for the linear mixed-effects models can be found in Table 2.

**Table 2:** Results from linear mixed-effects analysis

Predictors	Estimates	Std. Error	95% CI	Std. Coeff	Std. 95% CI	*p*
Depressive Symptoms						
Intercept	1.515	0.047	1.423–1.607	−0.511	−0.662 to −0.360	<0.001**
Sample [Current-Sample]	0.474	0.049	0.379–0.570	1.050	0.859–1.241	<0.001**
Grade	0.032	0.004	0.024–0.040	0.072	0.054–0.091	<0.001**
Sex [Female]	0.145	0.014	0.117–0.173	0.295	0.237–0.352	<0.001**
Parental education	−0.040	0.008	−0.056 to −0.024	−0.081	−0.113 to −0.048	<0.001**
Sample × Grade	0.032	0.008	0.017–0.046	0.071	0.038–0.105	<0.001**
Marginal R^2^/Conditional R^2^	0.239/0.582					
Social Anxiety						
Intercept	1.812	0.039	1.735–1.889	−0.287	−0.369 to −0.205	<0.001**
Sample [Current-Sample]	0.241	0.030	0.183–0.300	0.591	0.486–0.697	<0.001**
Grade	−0.013	0.005	−0.023 to −0.003	−0.027	−0.048 to −0.007	0.009*
Sex [Female]	0.089	0.017	0.056–0.122	0.168	0.106–0.231	<0.001**
Parental education	−0.038	0.010	−0.057 to −0.019	−0.071	−0.106 to −0.035	<0.001**
Sample × Grade	0.052	0.009	0.035–0.069	0.109	0.073–0.145	<0.001**
Marginal R^2^/Conditional R^2^	0.076/0.480					

Sex and Sample were coded as factors (0 = male, 1 = female; 0 = Past-Sample and 1 = Current-Sample). Grade was coded as numeric (0 to 3). Parental education was used as a proxy for SES. Bonferroni correction *p* < 0.025

*p < 0.025; **p < 0.001

### Depressive Symptoms

**Fig. 3:**
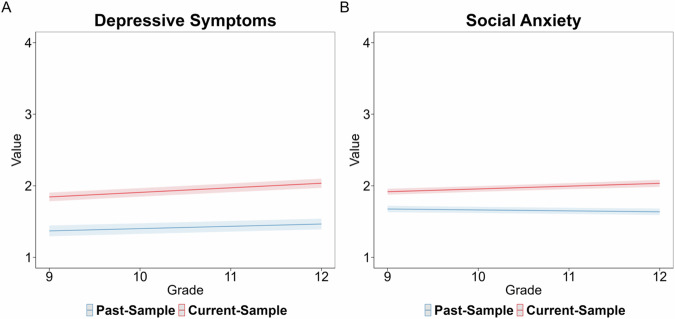
Effects plots for linear mixed-effects models for generational differences. Note. Effects plots for the grade-related differences in depressive symptoms (**A**) and social anxiety (**B**) across samples. The solid-colored lines represent the model fit. The shaded color regions depict the 95% confidence interval (CI)

A mixed-effects regression revealed that being in the Current-Sample (*B* = 0.474, *SE* = 0.049, *p* < 0.001), grade (*B* = 0.032, *SE* = 0.004, *p* < 0.001), being female (*B* = 0.145, *SE* = 0.014, *p* < 0.001), lower SES (*B* = −0.040, *SE* = 0.008, *p* < 0.001), and the interaction between sample and grade (*B* = 0.032, *SE* = 0.008, *p* < 0.001) significantly predicted depressive symptoms. The interaction indicated that the Current-Sample had higher depressive symptoms that increased at a steeper rate across grade compared to the Past-Sample, which increased at a less steep rate. The effects plot for the interaction is presented in Fig. 3A.

### Social Anxiety

A mixed-effects regression revealed that being in the Current-Sample (*B* = 0.241, *SE* = 0.030, *p* < 0.001), grade (*B* = −0.013, *SE* = 0.005, *p* = 0.009), being female (*B* = 0.089, *SE* = 0.017, *p* < 0.001), lower SES (*B* = −0.038, *SE* = 0.010, *p* < 0.001), and the interaction between sample and grade (*B* = 0.052, *SE* = 0.009, *p* < 0.001) significantly predicted social anxiety. The interaction indicated that the Current-Sample had higher social anxiety that increased at a steeper rate across grade compared to the Past-Sample, which decreased slightly across grade. The effects plot for the interaction is presented in Fig. 3B.

### Sex Differences Across High School Between Samples

The current study also assessed whether differences in mental health trends across high school between two generations of adolescents differed by sex. After correcting for multiple comparisons, the 3-way interaction was not significant in any of the models (*p*s < 0.025), suggesting that the pattern of results across generations is similar for males and females. See Supplementary Materials for full results.

**Research question 2: Are there generational differences in the proportion of adolescents at risk for consistently elevated mental health problems?**

Table 3 shows the proportion of adolescents with varying levels of elevated risk over time. Descriptively, the vast majority of adolescents in both the Past-Sample and Current-Sample did not report elevated mental health problems at any time points (72–88%). Across both samples, 9–19% of adolescents reported elevated mental health problems at one time point, indicating that they may be experiencing situational mental health problems that do not persist over time. Fewer adolescents (2–15%) reported elevated mental health problems at multiple time points, indicating that these adolescents may be at risk for experiencing chronic mental health problems.

**Table 3:** Proportion of Adolescents with Elevated Risk of Mental Health Problems Over Time

	Elevated Risk Over Time	Past-Sample (*n* = 1211)	Current-Sample (*n* = 857)
Depressive symptoms	No risk (0% of time points)	87.86%	74.33%
	Low risk (25–33.3% of time points)	10.16%	14.70%
	Moderate risk (50–66.7% of time points)	1.24%	8.28%
	High risk (>=75% of time points)	0.74%	2.68%
Social anxiety	No risk (0% of time points)	76.05%	72.00%
	Low risk (25–33.3% of time points)	19.32%	12.49%
	Moderate risk (50–66.7% of time points)	3.47%	9.80%
	High risk (>=75% of time points)	1.16%	5.72%

Proportion scores were calculated by summing the number of times an adolescent scored >= 2.5 (equivalent to often, always) on each mental health indicator, then dividing by the number of times each adolescent was present in the study. This method accounts for incomplete data. Only adolescents who had the opportunity to complete the survey more than once were included in this analysis; that is, 224 participants were removed from the Current-Sample for this analysis because they did not have the opportunity to complete the survey more than once (no participants were removed from the Past-Sample for this analysis)

A binomial regression was conducted to assess whether there were sample differences in the proportion of consistently at-risk adolescents across high school grades for depressive symptoms and social anxiety, controlling for sex and SES. In both models, there was a significant main effect of sample, such that the odds of being consistently at risk for depressive symptoms across the high school years were 3.64 times larger in the Current-Sample compared to the Past-Sample (*p* < 0.001), and the odds of being consistently at risk for social anxiety across the high school years were 2.25 times larger in the Current-Sample compared to the Past-Sample (*p* < 0.001). Full parameter estimates for the binomial regression models can be found in Table 4.

**Table 4:** Results from binomial regression analysis

Predictors	OR	Std. Error	95% CI for OR	*p*
Depressive Symptoms				
Intercept	0.147	0.039	0.087–0.249	<0.001**
Sample [Current-Sample]	3.636	0.419	2.907–4.567	<0.001**
Sex [Female]	2.139	0.217	1.757–2.614	<0.001**
Parental education	0.860	0.052	0.763–0.967	0.013*
Social Anxiety				
Intercept	0.229	0.048	0.152–0.346	<0.001**
Sample [Current-Sample]	2.249	0.203	1.886–2.685	<0.001**
Sex [Female]	1.391	0.109	1.194–1.623	<0.001**
Parental education	0.883	0.041	0.805–0.968	0.008*

Sex and Sample were coded as factors (0 = male, 1 = female; 0 = Past-Sample and 1 = Current-Sample). Parental education was used as a proxy for SES. Bonferroni correction *p* < 0.025

*OR* odds ratio

*p < 0.025; **p < 0.001

## Discussion

There has been extensive concern expressed from researchers, media, practitioners, and parents that youth from the current generation are facing a mental health crisis. The goal of this study was to assess whether there are generational trends in mental health problems across high school between adolescents today and adolescents from a previous generation 20 years ago. The current study used a longitudinal time-lag design to assess whether average levels of mental health problems and the proportion of adolescents consistently at risk for mental health problems over time were higher among adolescents in the current generation compared to adolescents from a previous generation. Overall, the current study found that adolescents from the current generation report higher mental health problems across the high school years, and more consistently elevated mental health problems, compared to adolescents from a previous generation.

The results indicated that adolescents in the Current-Sample reported on average, higher depressive symptoms and social anxiety that increased at a steeper rate across high-school compared to adolescents in the Past-Sample. It is important to note, however, that the average scores in both samples were around a “sometimes” on the scales, indicating that despite these differences, both samples showed relatively low mental health problems. Additionally, most adolescents in both samples did not report consistently elevated mental health problems across time. The Current-Sample, however, did have a higher proportion of adolescents who were classified as consistently at risk compared to the Past-Sample, indicating that there may be a small, yet growing, group of adolescents at risk for experiencing a “mental health crisis”.

There was no evidence of sex differences in the trends in mental health across samples in the current study. This finding suggests that the pattern of results across generations is similar for males and females. Females, however, had higher mental health problems than males across both samples. This finding is consistent with previous research suggesting that females may have an increased preponderance for mental health problems compared to their male counterparts (Anniko et al., 2019; Campbell et al., 2021; Morken et al., 2023). While this does not appear to be a generational phenomenon, attention should be given to females specifically as they may be most at risk for experiencing mental health problems during adolescence.

An important contribution of the current study was to assess differences in the proportion of students at risk for consistently high mental health problems in each sample. Most adolescents in both samples were never at an elevated risk for mental health problems, but there was a higher proportion of adolescents at risk for consistently elevated mental health problems across high school in the Current-Sample (10.96% and 15.52% were classified as moderate or high risk for depressive symptoms and social anxiety, respectively) compared to the Past-Sample (1.98% and 4.63% were classified as moderate or high risk for depressive symptoms and social anxiety, respectively). These adolescents are of particular concern. Interventions and resources aimed at improving adolescent mental health would benefit from directly targeting these adolescents who are consistently at risk over time. These findings also highlight the importance of using a longitudinal approach to understand generational differences among adolescents. Some adolescents may report elevated mental health problems at one time point, but then report lower mental health problems at later time points, indicating that their mental health problems may be situational and not indicative of a chronic problem.

There may be multiple reasons as to why the current generation experiences greater and more consistently high mental health problems across high school compared to a previous generation. The recent COVID-19 pandemic has widely been attributed to a rapid decline in adolescent mental health (Wolf & Schmitz, 2023). The results from the current study do not support this claim. There were no pandemic-related differences in depressive symptoms in the Current-Sample. There was a significant interaction between pandemic and grade for social anxiety, such that social anxiety was higher in grade 9 during non-pandemic years compared to during pandemic years. This finding makes sense given that adolescents entering high school in-person (i.e., during a non-pandemic year) may experience more nervousness in novel social situations compared to adolescents who entered high school online (i.e., during the pandemic). This finding also supports recent work suggesting that the pandemic was not a sole contributor to elevated mental health problems (Lebrun-Harris et al., 2022). Of course, the findings from the current study do not mean the pandemic did not impact adolescents’ mental health, as there may be important individual differences or subgroups of adolescents who experienced a differential impact of the pandemic (e.g., Borg & Willoughby, 2024).

Some researchers suggest that adolescents today may feel increasingly comfortable reporting mental health problems than in the past, given the decreased societal stigma and increased awareness efforts (e.g., Foulkes & Andrews, 2023), which may explain elevated levels of mental health problems. A concern raised by Foulkes and Andrews (2023) is that awareness efforts may lead some adolescents to potentially misinterpret mild or normative forms of distress as more clinical mental health problems (referred to by the authors as the Prevalence Inflation Hypothesis). This is an interesting idea that requires more direct testing.

In line with the Prevalence Inflation Hypothesis, it also is possible that there may be generational differences in how adolescents interpret their experiences with symptoms of mental health problems. In the current study, tests of measurement invariance suggested that the depressive symptoms and social anxiety constructs may not be measured equivalently across samples (though it is important to note that several researchers have suggested that this likely is nonproblematic for group comparisons; see Method section for more detail). There may be many reasons for these potential generational differences in interpretations of mental health measures. For example, educational programs targeted towards mental health literacy have been developed in recent years (Nobre et al., 2021) and as such, adolescents from more recent generations may have a more in-depth understanding of mental health problems and a heightened ability to detect symptoms. Future research should directly investigate whether there are generational differences in the interpretation of mental health measures.

Perhaps the most widely debated explanation attributed to mental health problems among youth today is technology and social media use (e.g., Odgers & Jensen, 2020). Technology use has changed rapidly over the last 20 years and thus, generational comparisons among adolescents requires careful consideration. Although beyond the scope of the current investigation, future work should critically and comprehensively assess the association between technology and mental health among adolescents. Recently, an advisory committee from the American Psychological Association published a report highlighting that a better understanding of the relationship between social media use and mental health among adolescents today requires careful consideration of the “content, features and function” of technology use (American Psychological Association, 2024). This is an exciting and important direction for future research.

The current study has several strengths, including data from two large longitudinal studies of adolescents from two generations using the same measures of mental health, and an assessment of mental health over the high school years. The samples were drawn from the same high schools, roughly 20 years apart, allowing for a direct comparison of adolescents from the same geographical region. The current study, however, is not without limitations. First, while a strength of the study is that the samples were drawn from the same high schools, this also is a limitation in that the results may be less generalizable to different regions. Additionally, the measures used in the current study did not include the full scale. The data were part of larger studies assessing a wide range of attitudes and behaviors, and thus, it was not feasible to include full scales for each measure. Overall, though, the measures used in the current study demonstrated good reliability.

## Conclusion

Research has lacked a longitudinal time-lag comparison of adolescents across the high school years from two generations to assess whether adolescents today are indeed experiencing a “mental health crisis”. The current study used data from two samples of adolescents from the same high schools, 20 years apart, to examine trajectories and consistency of depressive symptoms and social anxiety over time. Together, the findings from the current study suggest that on average, adolescents from the current generation report higher (but still relatively low) mental health problems across high school, and more consistently elevated levels of mental health problems over time, compared to a previous generation. Studies that use a cross-sectional time-lag design may miss out on the nuanced details about consistency over time, which requires the use of longitudinal data. Special attention should be paid to adolescents today who *consistently* report mental health problems over time, as they may be most at risk for negative outcomes associated with poorer mental health.

## Supplementary information

supplemental_materials

## Footnotes

1 All analyses were re-run without the cohort of adolescents who entered grade 9 in 2022 in the Current-Sample (i.e., adolescents who only were present at one time point; *n* = 224). The pattern of results remained the same. Thus, to preserve as much data as possible, this cohort of adolescents was kept in the Current-Sample.

2 To assess the measurement invariance of the depressive symptoms and social anxiety constructs between samples across the high school years, a series of confirmatory factor analyses were conducted. Configural, metric, and scalar invariance were tested. Sex and parental education were included in the models as covariates. The results did not support the assumption of measurement invariance (all chi-square difference tests *p* < 0.001); however, Putnick and Bornstein (2016) suggest that “A failure to demonstrate invariance should not necessarily preclude all further analyses of group or developmental differences. Non-invariance can be informative and may lead researchers to important conclusions about how different groups interpret the same construct” (p. 19) (see also Robitzsch and Lüdtke, 2022). Further, the measures used in the current study in both samples are validated measures that have been used in a variety of studies on adolescents over the past 30 years. Implications of measurement non-invariance in the current study are explored further in the discussion.
