# Supplementary material for: Generational Shifts in Adolescent Mental Health: A Longitudinal Time-Lag Study
Source: J Youth Adolesc. 2024 Oct 12;54(4):837–48. doi: 10.1007/s10964-024-02095-3 (PMC11933222; doi:10.1007/s10964-024-02095-3)
Supplement: Supplementary file 1 — supplemental_materials [file 10964_2024_2095_MOESM1_ESM.docx]

**Generational Shifts in Adolescent Mental Health: A Longitudinal Time-Lag Study**

Supplemental Table 1.

|  | Depressive Symptoms | | | |
| --- | --- | --- | --- | --- |
| *Predictors* | *Estimates* | *std. Error* | *CI* | *p* |
| Intercept | 1.511 | 0.049 | 1.416 – 1.607 | **<0.001**** |
| Cohort [Current-Sample] | 0.440 | 0.053 | 0.336 – 0.544 | **<0.001**** |
| Sex [Female] | 0.159 | 0.023 | 0.114 – 0.203 | **<0.001**** |
| Grade | 0.053 | 0.006 | 0.042 – 0.065 | **<0.001**** |
| Parental Education | -0.041 | 0.008 | -0.057 – -0.025 | **<0.001**** |
| Cohort × Sex | 0.072 | 0.034 | 0.007 – 0.138 | **0.031** |
| Cohort × Grade | 0.022 | 0.011 | 0.001 – 0.043 | **0.043** |
| Sex × Grade | -0.042 | 0.008 | -0.059 – -0.026 | **<0.001**** |
| Cohort × Sex × Grade | 0.021 | 0.015 | -0.007 – 0.050 | 0.143 |
| Marginal R^2^ / Conditional R^2^ | 0.243 / 0.585 | | | |

*Note.* Sex and Sample were coded as factors (0 = male, 1 = female; 0 = Past-Sample and 1 = Current-Sample). Grade was coded as numeric (0 to 3). SES was used as a proxy for parental education. Bonferroni correction *p* < .025.

* *p* < .025; ** *p* < .001.

Supplemental Table 2.

|  | Social Anxiety | | | |
| --- | --- | --- | --- | --- |
| *Predictors* | *Estimates* | *std. Error* | *CI* | *p* |
| Intercept | 1.841 | 0.041 | 1.760 – 1.923 | **<0.001**** |
| Cohort [Current-Sample] | 0.160 | 0.037 | 0.087 – 0.234 | **<0.001**** |
| Sex [Female] | 0.041 | 0.027 | -0.011 – 0.093 | 0.124 |
| Grade | 0.001 | 0.007 | -0.013 – 0.014 | 0.908 |
| Parental Education | -0.039 | 0.010 | -0.058 – -0.021 | **<0.001**** |
| Cohort × Sex | 0.166 | 0.039 | 0.089 – 0.244 | **<0.001**** |
| Cohort × Grade | 0.040 | 0.013 | 0.015 – 0.064 | **0.002*** |
| Sex × Grade | -0.027 | 0.010 | -0.047 – -0.008 | **0.005*** |
| Cohort × Sex × Grade | 0.025 | 0.017 | -0.009 – 0.059 | 0.149 |
| Marginal R^2^ / Conditional R^2^ | 0.084 / 0.481 | | | |

*Note.* Sex and Sample were coded as factors (0 = male, 1 = female; 0 = Past-Sample and 1 = Current-Sample). Grade was coded as numeric (0 to 3). SES was used as a proxy for parental education. Bonferroni correction *p* < .025.

* *p* < .025; ** *p* < .001.
